# The complete mitochondrial genome of Min pig (Hebao) and a phylogenetic analysis

**DOI:** 10.1080/23802359.2019.1678424

**Published:** 2019-10-21

**Authors:** Tianfu Niu, Liqun Yu, Xinmiao He, Shuai Zhao, Chunzhu Xu

**Affiliations:** a College of Life Science, Northeast Agricultural University, Harbin, PR China;; b Institute of Animal Husbandry, Heilongjiang Academy of Agricultural Sciences, Harbin, PR China

**Keywords:** Min pig (Hebao), mitochondrial genome, phylogeny

## Abstract

The complete mitochondrial genomes of Min pig (Hebao) are published in this paper. The full length of mtDNA is 16,719 bp and contained 13 PCGs, 2 rRNA and 22 tRNA, and 1D-loop(MN258706). A phylogenetic tree with the 13 protein-coding genes sequences of Min pig (Hebao) together with 45 other Chinese pig breeds and 7 foreign pig breeds was constructed. The results can be subsequently used to provide information for pig phylogenetic and insights into the evolution of genomes. The result of phylogenetic analysis showed that the genetic relationship of Min pig (Hebao) is closer to that of Dapulian pigs.

Min pig is an ancient local pig breed in Northeast China, which is mainly distributed in Northeast China and North China. Min pig has the characteristics of strong cold resistance, strong physique, strong fat deposition ability and good meat quality. It is an excellent variety of both meat and fat (Wang et al. 2011). The DNA samples from Min pig (Hebao) (NEAU2018-0704) come from the seed-holding farm of the pig in Institute of Animal Husbandry of Heilongjiang Academy of Agricultural Sciences, China in 2018 (45.6916N, 126.6263E). NEAU2018-0704 samples were deposited at museum of zoology, Northeast Agricultural University, China. The complete mitochondrial genome of Min pig (Hebao, GenBank accession no. MN258706) was amplified using polymerase chain reaction with 25 pairs of primers designed according to mitogenome of the Wuzhishan pig (KF767443) (Yu et al. 2015). DNAMAN v6.0.3.99 was used to proofread and splice the sequencing results, and the base composition, content and full length of the whole sequence of mtDNA was counted. Use Chromas v2.4.1 software and DNASTAR v7.1 software to comment and splice.

As a result, the mitochondrial genome of the pig is 16, 719 bp in length and including 13 protein-coding genes (PCGs), 2 rRNA genes, and 22 tRNA genes. Eleven of the 13 PCGs (ND1, NAD3, NAD4, COX1, COX2, N8, ATP6, COX3, CYTB) used ATG as the starting codon, another one (ND4L) used the ATA and (ND2, ND3, ND5) used GTG. Twelve genes (ND1, ND2, COX2, ND6, COX1, COX3, ND3, ATP6, NAD4L, and NAD5) end with a TAA stop codon, but for them (ND1, ND2, COX2, COX3, NAD3), TAA stop codon is completed by the addition of 3’A residues to the mRNA, and ATP8 gene was terminated with the TAG stop codon. CYTB and ND4 end with TGA stop codon, but the stop codon of ND4 is complete by the addition of 3′A residues to the mRNA.

To assess the genetic relationship among different pig breeds in China, phylogenetic analysis was performed using the complete mitochondrial DNA sequences of 52 pig breeds. The phylogenetic analysis of 13 protein-coding gene sequences was performed. The sequence of all types of pigs was calibrated, cut and aligned using BioEdit v7.0.9 (Alzohairy 2011), Resulting in 11,409 bp length alignment. The phylogenetic relationships were reconstructed using the maximum likelihood (ML) method in the MEGA X v10.0.2 (Kumar et al. 2018). The best substitution model was chosen in the jModelTest v2.1.10 (Darriba et al. 2012) based on the Bayesian information criterion (BIC). According to JModelTest, the best model describing the evolution of the mitogenomes, and therefore, subsequent ML analysis and results (Figure 1). Min pig (Hebao) is closely related to Dapulian pig, which may be different from the traditional regional classification, and needs to study further. The three foreign breeds of Vietnam, Korean, and Russia are far apart from Chinese cluster distribution. Among them, the Min pig (Hebao) studied in this study were gathered together with Dapulian pigs. They are all North China pigs (Wang et al. 2011). According to the tree diagram, the genetic relationship of Min pig (Hebao) is closer to that of Dapulian pigs.

**Figure 1.**
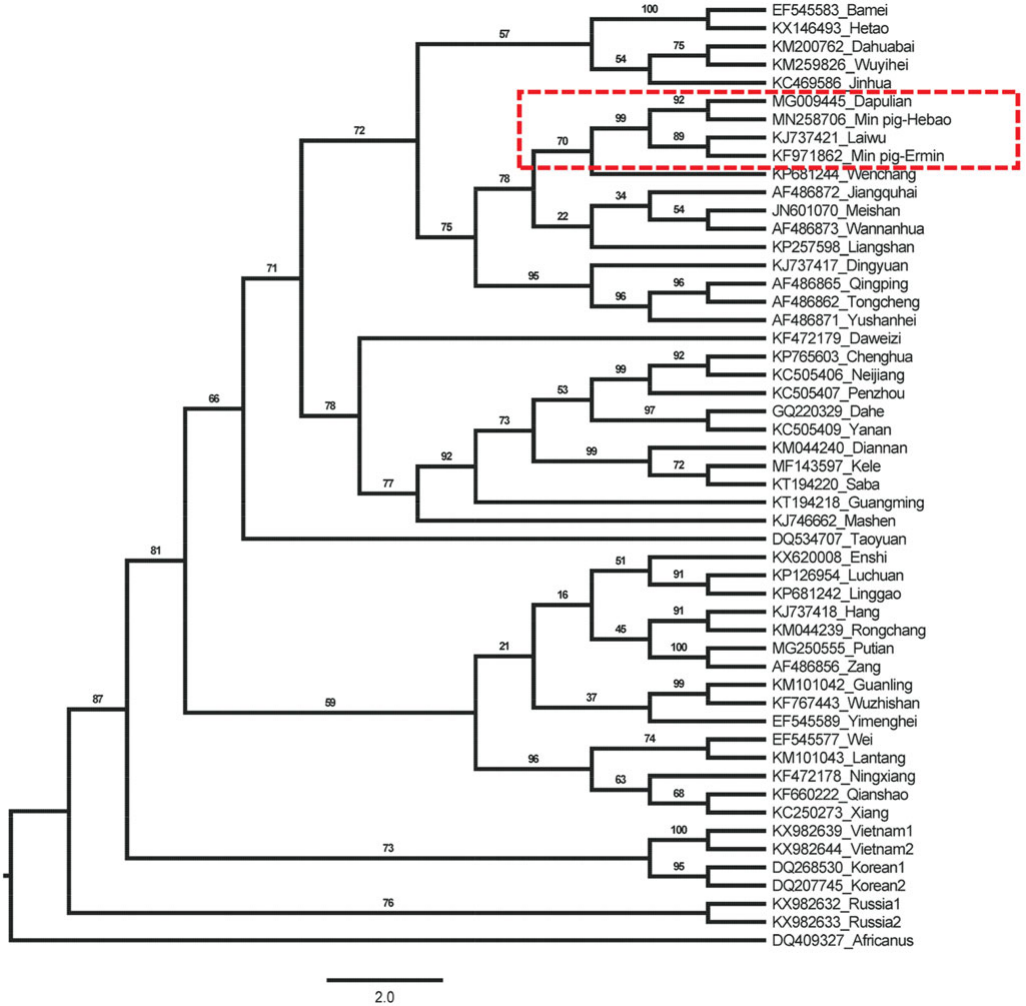
The maximum-likelihood phylogenetic tree for different kinds of pigs, the phylogenetic analysis of 13 PCG sequences was performed.

## References

[CIT0001] Alzohairy AM 2011 Bioedit: an important software for molecular biology. Gerf Bull Biosci. 2:60–61.

[CIT0002] Darriba D, Taboada GL, Doallo R, Posada D 2012 Jmodeltest 2: more models, new heuristics and parallel computing. Nat Methods. 9:772–772. 10.1038/nmeth.2109 PMC4594756 22847109

[CIT0003] Kumar S, Stecher G, Li M, Knyaz C, Tamura K 2018 Mega x: molecular evolutionary genetics analysis across computing platforms. Mol Biol Evol. 35:1547–1549. 29722887 10.1093/molbev/msy096 PMC5967553

[CIT0004] Wang CY, Wang AG, Wang LX, Li K, et al. 2011 Animal genetic resources in China pigs. China Agric Ture Press. 5:25–29.

[CIT0005] Yu P, Cao T, Zhao CP, Liu YF, Shi LG, Zhang LL, Hou GY 2015 Complete mitochondrial genome sequence and analysis of Wuzhishan Pig. Acta Agriculturae Boreali – Occidentalis Sinica. 24:16–22.

